# Supplementary material for: Genome-wide identification, characterization and gene expression of BES1 transcription factor family in grapevine (Vitis vinifera L.)
Source: Sci Rep. 2023 Jan 5;13:240. doi: 10.1038/s41598-022-24407-y (PMC9816167; doi:10.1038/s41598-022-24407-y)
Supplement: Supplementary file 3 — Supplementary Information. [file 41598_2022_24407_MOESM3_ESM.zip › Vvi_Atr/Vitis_vinifera.PN40024.v4.dna_sm.toplevel.fa.vs.Amborella_trichopoda.AMTR1.0.dna_sm.toplevel.fa.html/Atr-AmTr_v1.0_scaffold00166.html]

|  |  |  |  |  |  |  |  |  |  |  |  |  |  |
| --- | --- | --- | --- | --- | --- | --- | --- | --- | --- | --- | --- | --- | --- |
| Duplication depth | Reference chromosome | Collinear blocks | | | | | | | | | | | |
| 0 | Atr-ERN10503 |  |  |  |  |  |  |  |  |  |  |  |  |
| 0 | Atr-ERN10504 |  |  |  |  |  |  |  |  |  |  |  |  |
| 0 | Atr-ERN10505 |  |  |  |  |  |  |  |  |  |  |  |  |
| 0 | Atr-ERN10506 |  |  |  |  |  |  |  |  |  |  |  |  |
| 0 | Atr-ERN10507 |  |  |  |  |  |  |  |  |  |  |  |  |
| 1 | Atr-ERN10508 |  | Vvi-Vitvi17g01581\_t001 |  |  |  |  |  |  |  |  |  |  |
| 1 | Atr-ERN10509 |  | Vvi-Vitvi17g00964\_t001 |  |  |  |  |  |  |  |  |  |  |
| 1 | Atr-ERN10510 |  | \| \| |  |  |  |  |  |  |  |  |  |  |
| 1 | Atr-ERN10511 |  | Vvi-Vitvi17g00963\_t001 |  |  |  |  |  |  |  |  |  |  |
| 1 | Atr-ERN10512 |  | \| \| |  |  |  |  |  |  |  |  |  |  |
| 1 | Atr-ERN10513 |  | \| \| |  |  |  |  |  |  |  |  |  |  |
| 1 | Atr-ERN10514 |  | \| \| |  |  |  |  |  |  |  |  |  |  |
| 1 | Atr-ERN10515 |  | \| \| |  |  |  |  |  |  |  |  |  |  |
| 1 | Atr-ERN10516 |  | Vvi-Vitvi17g00959\_t001 |  |  |  |  |  |  |  |  |  |  |
| 1 | Atr-ERN10517 |  | \| \| |  |  |  |  |  |  |  |  |  |  |
| 1 | Atr-ERN10518 |  | \| \| |  |  |  |  |  |  |  |  |  |  |
| 1 | Atr-ERN10519 |  | \| \| |  |  |  |  |  |  |  |  |  |  |
| 1 | Atr-ERN10520 |  | \| \| |  |  |  |  |  |  |  |  |  |  |
| 1 | Atr-ERN10521 |  | \| \| |  |  |  |  |  |  |  |  |  |  |
| 1 | Atr-ERN10522 |  | \| \| |  |  |  |  |  |  |  |  |  |  |
| 1 | Atr-ERN10523 |  | \| \| |  |  |  |  |  |  |  |  |  |  |
| 1 | Atr-ERN10524 |  | \| \| |  |  |  |  |  |  |  |  |  |  |
| 1 | Atr-ERN10525 |  | \| \| |  |  |  |  |  |  |  |  |  |  |
| 1 | Atr-ERN10526 |  | \| \| |  |  |  |  |  |  |  |  |  |  |
| 1 | Atr-ERN10527 |  | \| \| |  |  |  |  |  |  |  |  |  |  |
| 1 | Atr-ERN10528 |  | \| \| |  |  |  |  |  |  |  |  |  |  |
| 1 | Atr-ERN10529 |  | \| \| |  |  |  |  |  |  |  |  |  |  |
| 1 | Atr-ERN10530 |  | \| \| |  |  |  |  |  |  |  |  |  |  |
| 1 | Atr-ERN10531 |  | \| \| |  |  |  |  |  |  |  |  |  |  |
| 1 | Atr-ERN10532 |  | \| \| |  |  |  |  |  |  |  |  |  |  |
| 1 | Atr-ERN10533 |  | \| \| |  |  |  |  |  |  |  |  |  |  |
| 1 | Atr-ERN10534 |  | \| \| |  |  |  |  |  |  |  |  |  |  |
| 1 | Atr-ERN10535 |  | \| \| |  |  |  |  |  |  |  |  |  |  |
| 1 | Atr-ERN10536 |  | \| \| |  |  |  |  |  |  |  |  |  |  |
| 1 | Atr-ERN10537 |  | \| \| |  |  |  |  |  |  |  |  |  |  |
| 1 | Atr-ERN10538 |  | Vvi-Vitvi17g00958\_t001 |  |  |  |  |  |  |  |  |  |  |
| 1 | Atr-ERN10539 |  | \| \| |  |  |  |  |  |  |  |  |  |  |
| 1 | Atr-ERN10540 |  | \| \| |  |  |  |  |  |  |  |  |  |  |
| 1 | Atr-ERN10541 |  | \| \| |  |  |  |  |  |  |  |  |  |  |
| 1 | Atr-ERN10542 |  | Vvi-Vitvi17g04271\_t001 |  |  |  |  |  |  |  |  |  |  |
| 1 | Atr-ERN10543 |  | Vvi-Vitvi17g00955\_t001 |  |  |  |  |  |  |  |  |  |  |
| 1 | Atr-ERN10544 |  | Vvi-Vitvi17g00954\_t001 |  |  |  |  |  |  |  |  |  |  |
| 1 | Atr-ERN10545 |  | \| \| |  |  |  |  |  |  |  |  |  |  |
| 1 | Atr-ERN10546 |  | \| \| |  |  |  |  |  |  |  |  |  |  |
| 1 | Atr-ERN10547 |  | Vvi-Vitvi17g00953\_t001 |  |  |  |  |  |  |  |  |  |  |
